# Brain Aging and Cellular Senescence Trigger Neuroimmunological Dysregulation and Neurodegeneration

**DOI:** 10.3390/brainsci16101019

**Published:** 2026-09-24

**Authors:** Vega-García Angélica, Manuel-Apolinar Leticia, Feria-Romero Iris Angélica, Campos-Bedolla Patricia, Gutiérrez-García Carlos Alberto, Luna-Munguía Hiram, Reyes-Huesca Sofía, Orozco-Suárez Sandra

**Affiliations:** 1 Unidad de Investigación Médica en Enfermedades Neurológicas, Hospital de Especialidades, “Dr. Bernardo Sepúlveda”, Centro Médico Nacional Siglo XXI, Instituto Mexicano del Seguro Social, Ciudad de México 06720, CP, Mexico; ange_li_k@hotmail.com (V.-G.A.); iris.feria@imss.gob.mx (F.-R.I.A.); camposbedollap@hotmail.com (C.-B.P.); dr.carlosgutierrezt@gmail.com (G.-G.C.A.); sofia_reyesh@yahoo.com.mx (R.-H.S.); 2 Unidad de Investigación Médica en Enfermedades Endocrinas, Hospital de Especialidades, “Dr. Bernardo Sepúlveda”, Centro Médico Nacional Siglo XXI, Instituto Mexicano del Seguro Social, Ciudad de México 06720, CP, Mexico; leticia.manuel@imss.gob.mx; 3 Programa de Maestría en Ciencias de la Salud, Sección de Estudios de Posgrado e Investigación de la Escuela Superior de Medicina, Instituto Politécnico Nacional, Ciudad de México 11340, CP, Mexico; 4 Laboratorio de Conectividad Cerebral, Departamento de Neurobiología Conductual y Cognitiva, Instituto de Neurobiología, UNAM, Campus Juriquilla, Querétaro 76230, CP, Mexico; hiram_luna@inb.unam.mx; 5 Programa de Doctorado en Investigación en Medicina, Sección de Estudios de Posgrado e Investigación de la Escuela Superior de Medicina, Instituto Politécnico Nacional, Ciudad de México 11340, CP, Mexico

**Keywords:** neuroimmune dysregulation, immunosenescence, neuroinflammation, brain aging, neurodegeneration

## Abstract

**Highlights:**

**What are the main findings?**

- The aging process is heterogeneous and marked by epigenetic alterations that promote cellular senescence, activate SASP, and trigger a cascade of signaling pathways, including cGAS-STING, C/EBPβ, NF-κB, and mTOR, which converge into a vicious cycle of immunosenescence and inflammaging.
- BBB damage from inflammaging alters astrocyte and microglial function, inducing morphological changes that generate a proinflammatory microenvironment akin to SASP activation within the brain parenchyma, a phenomenon termed neuroinflammaging.

**What are the implications of the main findings?**

- Aging is a progressive, heterogeneous process in which the CNS exhibits a developmental lag relative to the aging of peripheral organs.
- Protecting the blood–brain barrier may be a key mechanism to prevent the continuous recruitment and entry of peripheral immune cells into the brain parenchyma and a way to modulate glial cell reactivity during neuroinflammaging.

**Abstract:**

Cellular senescence during brain aging is a complex biological phenomenon triggered by age-related and external factors, leading to epigenetic and genetic alterations that impair cellular replication, cause mitochondrial dysfunction, and activate the senescence-associated secretory phenotype (SASP). This activation initiates proinflammatory signaling cascades and impairs inflammation-resolution mechanisms, facilitating the accumulation of immunosenescent cells and proinflammatory mediators and promoting the chronic, age-associated inflammation known as “inflammaging.” Although immunosenescence and inflammaging are distinct mechanisms, they are not independent. Both can contribute to the development and progression of neurodegenerative disorders by maintaining a chronic systemic proinflammatory state mediated by dysfunction of signaling pathways involved in lipid metabolism and inflammation resolution, as well as by disrupting the blood–brain barrier, which facilitates neuroinflammation within the brain parenchyma. Furthermore, dysfunction in bidirectional communication pathways between the immune system and the central nervous system, driven by immunosenescence in neural cells, including glial cells and neurons, which are postmitotic cells that can acquire phenotypic traits associated with senescence, facilitates SASP activation, leading to neuroinflammation and neurodegeneration. This review examines the mechanisms underlying brain aging and SASP activation, as well as the signaling pathways involved in inflammaging and its links to immunosenescence, blood–brain barrier damage, and neurodegeneration, which together facilitate the development and progression of neurodegenerative diseases and pathological aging.

## 1. Introduction

Aging is a complex phenomenon marked by progressive, systemic physiological changes that are genetically regulated and shaped by environmental conditions throughout an individual’s life [1,2,3]. Current demographic projections indicate that by 2030, one in six people worldwide will reach age 60, and by 2050, the global population aged 80 or older will exceed 400 million, making aging a significant public health issue [4].

In 1981, Denham Harman first defined aging as the progressive accumulation of cellular damage that increases susceptibility to various diseases, with onset in early old age, specifically at age 60 [5]. Recent research describes aging as a gradual, functional, and heterogeneous decline in tissue and organ function; this decline can be antagonistic, occurring in response to damage, such as cellular senescence, or result from activation of the aging phenotype by specific factors [2,3,6]. Furthermore, aging is described in terms of three periods: early old age (ages 60 to 75), characterized by the onset of a gradual and heterogeneous deterioration in the organism’s structure and function, observed as a progressive decline in physical capabilities; middle old age (ages 76 to 90), primarily characterized by a decline in motor capabilities; and finally, late old age, marked by a more pronounced deterioration of physical, motor, and cognitive capabilities [7]. 

To aid study and understanding, the complex and diverse molecular and cellular changes, including genomic instability, telomere shortening, epigenetic alterations, mitochondrial dysfunction, loss of proteostasis, impaired macroautophagy, nutrient-sensing dysregulation, stem cell exhaustion, and cellular senescence, have been described as “windows of aging”; these processes gradually alter intercellular communication across multiple organs and systems during physiological aging [2,3,8]. However, repeated exposure to pathogens, toxins, trauma, and chronic diseases can create feedback loops of cellular damage that trigger the senescence-associated secretory phenotype (SASP) and activate proinflammatory signaling pathways. At the same time, increased oxidative stress causes mitochondrial dysfunction and oxidative DNA damage, promoting immunosenescence and, consequently, age-associated inflammation [9,10,11,12].

This manifests through two distinct mechanisms: pathogen-driven non-sterile immune responses and aging-associated low-grade sterile inflammation commonly termed “inflammaging.” In turn, SASP activation within the immune system can lead to immunosenescence (primarily affecting B and T lymphocytes). This decline in innate and adaptive immune responses fosters a pro-inflammatory environment that compromises protective barriers in the central nervous system (CNS), such as the blood–brain barrier (BBB). Thereby allowing immune cells and proinflammatory molecules to enter the brain parenchyma [9,10,11,12]. Similarly, senescence in cerebral microcapillary brain endothelial cells (MBECs), astrocytes, microglia, and neurons, which are postmitotic cells that can acquire phenotypic traits associated with senescence, leads to irreversible cell cycle arrest and SASP activation, creating an age-associated neurogenic inflammatory state known as “neuroinflammaging” [2,3,4,5,6,11,12]. MBECs senescence and BBB damage can exacerbate both inflammaging and immunosenescence, disrupting bidirectional communication between the immune system and the CNS during aging [2,3,4,5,6].

This review focuses on the mechanisms underlying brain aging and SASP activation, as well as the signaling pathways involved in inflammaging and its links to immunosenescence, BBB damage, and neurodegeneration, which together facilitate the development and progression of neurodegenerative diseases and pathological aging.

## 2. Cellular Senescence in Aging

Cellular senescence is a state of cell-cycle arrest that occurs during the embryonic stage and persists throughout life as a preventive mechanism against cancer cell proliferation [13,14,15]; it also participates in tissue repair and promotes inflammatory responses during aging [16,17]. However, acute or chronic damage can also trigger cellular senescence [3,13]. It is characterized by prolonged, generally irreversible cell-cycle arrest, macromolecular damage, metabolic alterations, and the acquisition of a distinctive, activated SASP [3,13,17]. Senescent cells strongly stimulate immune cells and can trigger non-infectious systemic inflammation by actively releasing proinflammatory mediators and stress signals [18,19,20]. The immune system’s recognition and subsequent elimination of these senescent cells are vital for controlling inflammation, promoting tissue regeneration, and restoring normal function [20,21].

However, during aging, cellular senescence plays a central role in opposing mechanisms mediated by both exogenous and endogenous agents that lead to epigenetic changes, genomic instability, telomere shortening, single- and double-strand breaks, chromosomal rearrangements, and defects in nuclear architecture, all of which increase random variability in gene expression [22,23,24].

Furthermore, aberrant mRNA production and maturation have been reported to promote cellular dysfunction, inhibit macroautophagy and mitophagy, and thereby facilitate mitochondrial mutations and disrupt energy balance [23,24,25,26]. These processes also drive oxidative damage, degrade non-protein macromolecules, including DNA, lipid vesicles, organelles, and glycogen, and disrupt proteostasis [27,28,29,30]. Altered proteostasis destabilizes respiratory chain complexes, and reduced organelle turnover and altered mitochondrial dynamics compromise cellular bioenergetics [31]. Reactive oxygen species (ROS) contribute to mitochondrial dysfunction by increasing mitochondrial membrane permeability and promoting mitochondrial DNA release. Mitochondrial DNA (mitDNA) released into the cytosol is detected by the damage-associated molecular pattern (DAMP) program, where it acts as a DAMP that activates innate immunity and mediates the inflammatory response [32]. Likewise, increased cytoplasmic DNA (cytoDNA) is detected by cyclic GMP-AMP synthase (cGAS), which binds cytoDNA and generates cyclic GMP-AMP (cGAMP), which STING recognizes. Subsequently, STING activates the synthesis of type I interferons (IFNs) and proinflammatory cytokines by activating nuclear factor of kappa light chain enhancer (NFκ-B), thereby promoting a chronic inflammatory state associated with aging, known as inflammaging (Figure 1) [32,33,34].

### 2.1. Main Molecular Mechanisms of Cellular Senescence That Converge on Inflammaging

The progressive accumulation of senescent cells in various tissues during aging, primarily fibroblasts, MBECs, and immune cells in so-called postmitotic or slowly proliferating tissues such as the heart and brain [35,36], triggers the recruitment of phosphorylated histones, including γH2AX, which participate in DNA damage and repair signaling pathways [13,21] and are associated with chromatin alterations, disruptive remodeling of the nuclear lamina, decreased expression of laminin B1, and a progressive redistribution or loss of heterochromatin that leads to increased genomic instability and altered gene expression [21,37,38]. This process is mediated by the accumulation of micronuclei and cytoplasmic chromatin fragments, also known as cytDNA fragments [18,39,40,41,42].

This accumulation of cell-free cytDNA and circulating factors stimulates the SASP, a range of proinflammatory cytokines and chemokines, including interleukin (IL-1α), IL-6, IL-8, and tumor necrosis factor (TNF-α), as well as growth and angiogenic factors such as epidermal growth factor (EGF), vascular endothelial growth factor (VEGF), insulin-like growth factor-binding protein (IGFBP)-1, IGFBP-2, matrix metalloproteinases (MMP)-1, MMP-3, MMP-12, and tissue inhibitor of metalloproteinases 1 (TIMP-1) [18,43]. In addition, NF-κB regulates proinflammatory molecules, including CCAAT-binding protein β (C/EBPβ), bromodomain-containing protein 4 (BRD4), lysine methyltransferase MLL1, and euchromatic histone lysine methyltransferase 2 (EHMT2) [18,43].

SASP is a hallmark of senescent cells and is implicated in numerous pathophysiological mechanisms, including reinforcing and spreading senescence through autocrine and paracrine pathways, activating immune responses that eliminate senescent cells during development, promoting cellular plasticity, and promoting tumorigenesis through angiogenesis and metastasis [21,43,44,45]. However, persistent SASP secretion elevates proinflammatory mediators that impair the phagocytic function of resident macrophages, reducing their ability to eliminate senescent cells and to support tissue repair, causing genomic damage, disrupting proteostasis, and altering cellular metabolism, collectively perpetuating the inflammatory response [46,47].

These interconnected processes during aging contribute to macrophage polarization in tissue. Activated macrophages can differentiate into phenotypically distinct states, including classically activated macrophages (M1) and alternatively activated macrophages (M2). The proinflammatory M1 phenotype is driven by endoplasmic reticulum (ER) stress, reduced waste clearance via the proteasome, autophagy, and mitochondrial dysfunction [48,49]. This facilitates the accumulation of M1 macrophages in tissues, promoting inflammation and dysregulation of innate and adaptive immunity during aging [50,51,52]. The increase in exogenous stressors, such as blood–brain barrier dysfunction and gut dysbiosis, also contributes to age-related intrinsic changes in macrophages under different activation thresholds, which are triggered by increased damage to proteins, lipids, and nucleic acids, organelle dysfunction, and increased levels of Toll-like receptors (TLRs), receptors for advanced glycation end products (RAGEs), and S100A8/A9 proteins, which are upregulated in the systemic circulation [2,52,53].

Similarly, increased levels of the alpha subunit of hypoxia-inducible factor 1 (HIF-1α), a hypoxia-induced product, are linked to age-related vascular dysfunction, and NF-κB activation stimulates proinflammatory cytokine and ROS production [54]. Furthermore, aging is associated with an intrinsic failure to terminate the innate apoptotic immune response, accompanied by increased expression of TNF-α, IL-1β, and IL-6, and with immunosenescence [55]. This innate apoptotic immunity recognizes and responds to specific apoptotic cells, distinguishing dead from viable cells and immediately inducing anti-inflammatory cytokine gene transcription while inhibiting pro-inflammatory cytokine gene transcription in the responding cell [56]. Failure of this apoptotic immune response is associated with inflammatory and autoimmune diseases and immunosenescence [57,58,59]. Through the SASP, senescent cells not only generate a pro-inflammatory microenvironment but also propagate it to neighboring cells, establishing a positive feedback loop between inflammation and cellular senescence; however, the precise molecular mechanisms remain incompletely understood [21,43,44,45,46,47]. Therefore, the following section describes molecules activated within the SASP-mediated signaling cascade that converge on “inflammaging”.

### 2.2. SASP-Activated Molecular Signaling Promotes Inflammaging

#### 2.2.1. cGAS-STING

The cGAS-STING signaling pathway detects DNA damage and cytoDNA in senescent cells. STING is a dimeric transmembrane protein localized in the ER that orchestrates innate immune responses to 2′3′-cGAMP [60,61,62]. cGAS is the primary sensor of cytoDNA present outside subcellular compartments such as the nucleus and mitochondria [62]. Upon recognizing cytoDNA, cGAS dimerizes and stimulates the formation of cyclic GMP-AMP (cGAMP), which binds directly to the STING (stimulator of interferon genes) protein. This triggers TBK1-mediated phosphorylation and activation of the transcription factor IRF3, which induces interferon-stimulated genes (ISGs) [62,63]. The cGAS-STING pathway is the primary mechanism responsible for detecting cellular stress, DNA damage, and inflammation through the production of proinflammatory cytokines and type I interferon (IFN-I) in age-related diseases, autoimmune disorders, and neoplasms [64,65]. 

The cGAS-STING proteins are expressed in a variety of immune cells, including macrophages, dendritic cells, natural killer (NK) cells, T and B lymphocytes, BMECs, epithelial cells, and neurons [66]. cGAS-STING can promote or modulate cellular senescence [67,68,69]. STING activity has been linked to SASP activation, and its inhibition significantly reduces proinflammatory mediator expression in senescent cells [70]. STING activation is associated with exacerbated proinflammatory responses in macrophages in chronic inflammatory diseases, such as nonalcoholic fatty liver disease (NAFLD) and liver fibrosis [71,72,73]. In addition, STING signaling is associated with NF-κB activation [63,74], and disruption of cGAS-STING signaling contributes to senescence and SASP-derived inflammation (Figure 1) [52,75].

#### 2.2.2. C/EBPβ

Another important signaling pathway involves the transcription factor C/EBPβ (CCAAT-binding protein/beta enhancer-binding protein), which belongs to the C/EBP family and regulates immune responses and inflammation through the MAPK/NF-κB and IL-6/JAK/STAT3 signaling pathways. It is essential for macrophage activation and inflammatory cytokine expression [76,77]. C/EBPβ, also known as NF-IL6, binds to the IL-1β response element in the IL-6 promoter, promoting transcription of IL-6 and IL-8. Furthermore, C/EBPβ interacts directly with the Rel homology domain shared by the p50, p65, and c-Rel subunits, thereby stabilizing NF-κB and activating IL-6 and IL-8 expression [78]. In turn, C/EBPβ can directly inactivate IκBα, a protein that regulates NF-κB, thereby balancing anti-inflammatory and pro-inflammatory signals during tissue repair [78,79].

Therefore, C/EBPβ and NF-κB have been shown to promote cellular senescence and act as transcriptional co-regulators of the inflammatory response by constitutively inducing the SASP during aging, thereby maintaining a chronic inflammatory state [78,79,80,81]. Cappello et al. [58] observed that C/EBPβ stimulated NF-κB-dependent signaling by decreasing IκB-α expression an NF-κB inhibitor. The C/EBPβ gene is located on human chromosome 20q13.13 and the N-terminal region of LAP (the long isoform of C/EBPβ, also known as NF-IL6) contains 21 amino acids and mediates interaction with NF-κB [58,82,83]. Furthermore, C/EBPβ can interact with itself or with other members of the C/EBP family to form homodimers or heterodimers, respectively, thereby increasing C/EBPβ’s affinity for DNA. Conversely, reduced dimer formation significantly lowers C/EBPβ’s affinity for DNA [84,85]. In addition, proinflammatory cytokines such as IL-1β, IL-6, and TNF-α can activate C/EBPβ [86], induce phosphorylation of its transactivation domain (TAD), and, through a positive feedback loop, increase expression of C/EBPβ-mediated proinflammatory genes in response to these stimuli [87].

In macrophages, C/EBPβ has been shown to modulate IL-1β, IL-6, IL-8, TNF-α, granulocyte colony-stimulating factor (G-CSF), nitric oxide synthase (NOS), neutrophil elastase, lysozyme, and the granulocyte receptor. C/EBPβ positively regulates the gene expression of MIP-1α, osteopontin, and CD14 [87,88]. Furthermore, increased TNF-α levels facilitate the nuclear localization of C/EBPβ in response to inflammatory stress [89]. Although NF-κB positively regulates C/EBPβ mRNA expression, researchers have not identified a functional NF-κB binding site in the C/EBPβ promoter [90]. Nevertheless, interactions among C/EBPβ, the glucocorticoid receptor (GR), and the Jun and Fos factors [90,91,92] may regulate proinflammatory cytokine expression, such as IL-1β, in lipopolysaccharide (LPS)- treated astrocytes. Furthermore, the joint activation of AP-1, NF-κB, and C/EBPβ can induce activation of the NOS2 promoter [93,94,95].

#### 2.2.3. Transcription Factors NF-κB, TGF-β and HMGB1

NF-κB-mediated signaling pathways are the primary regulators of innate immunity and induce SASP and inflammation during cellular senescence [95,96]. Innate immune cells, such as macrophages, dendritic cells, and neutrophils, express pattern recognition receptors (PRRs) that detect microbial components known as pathogen-associated molecular patterns (PAMPs) and recognize molecules released by damaged or necrotic cells as DAMPs. TLRs, RIG-I-like receptors, NOD-like receptors, C-type lectin receptors (CLRs), and cytoplasmic DNA sensors primarily recognize these molecules [97,98,99].

NF-κB activation involves two main signaling pathways: the canonical and the non-canonical (or alternative) pathways, both of which regulate immune and inflammatory responses. Although they differ in their signaling mechanisms, the canonical NF-κB pathway induces, at the transcriptional level, the production of proinflammatory cytokines, chemokines, and inflammatory mediators that directly drive the differentiation of inflammatory T cells [100]. Similarly, transforming growth factor-β (TGF-β) activated kinase 1 (TAK1) and its two regulatory subunits, TAB1 and TAB2, which are part of different PRR pathways, converge on NF-κB activation: TAK1 activates IKK, which phosphorylates IκBα and thereby promotes NF-κB activation [100,101,102]. Another key regulator of the IKK complex is NEMO (NF-κB-modulating ubiquitin-binding protein), which interacts with NF-κB signaling proteins such as the ataxia telangiectasia mutated (ATM) protein, ELKS, RIP1, and TAK1 in response to DNA damage induced by oxidative stress [101,102,103]. NEMO-independent activation of the IKK complex can also occur via the ATM/TRAF6/cIAP1 pathway, through the TAK1-dependent IKK complex, which activates IKKβ and p38MAPK proteins that play a crucial role in the feedback loop between cellular senescence and age-related inflammation [103,104]. Furthermore, the TGF-β family of cytokines regulates various cellular functions mediated by SMAD pathways (intracellular proteins that act as second messengers), such as proliferation, differentiation, and apoptosis, as well as through non-SMAD pathways, namely TRAF6–TAK1–p38MAPK and TAK1 signaling pathways, which activate the IKK–NF-κB pathway and induce cell cycle arrest and senescence [105,106,107,108]. Notably, TGF-β signaling activity increases with aging, particularly in the brain [109], where it promotes microvascular degeneration and gliosis [110].

Additionally, TAK1 is involved not only in TGFβ signaling but also in regulating NF-κB activation induced by IL-1β and TNFα [111,112]. TAK1 interacts with TAK1-binding protein 1 (TAB1) and is activated by phosphorylation at Thr178, Thr187, and Thr184 within TAK1’s T-loop [104,112]. Autophosphorylation of TAK1 is necessary for IL-1β-mediated activation of the IKK–NF-κB and JNK-AP-1 signaling pathways, as well as for IL-6 expression [113]. The TAK1 complex associates with the adaptors TAB2, TAB3, and TAB4, which recruit other modulators, such as RIP1, NEMO, and MKK3/6, all of which activate pro-inflammatory signaling pathways [114]. Furthermore, Hsp90 targets TAK1, thereby stabilizing pro-inflammatory complexes [115].

In this context, the glycosylated protein TMEM9B, localized to lysosomal membranes and early endosomes, has been shown to contribute to NF-κB activation by TNF-α, IL-1β, and TLR ligands during inflammation [115,116]. TMEM9B interacts with RIP1 and TAK1, which stimulate pro-inflammatory signaling via the p38MAPK and JNK pathways, underscoring the importance of NF-κB signaling in SASP activation [115,116]. Furthermore, HMGB1, a member of the high-mobility non-histone chromatin-associated protein family, is involved in DNA transcription, replication, and repair [117]. HMGB1 acts as a proinflammatory cytokine in response to excessive cellular stress and the recruitment of immune cells to tissues; it is released through inflammasome activation and interacts with transcription factors, cytokines such as IL-1β and TNF-α [117,118,119], and innate immune receptors such as RAGE, TLR2, and TLR4 linked to the NF-κB pathway [102,120].

#### 2.2.4. mTOR and JAK/STAT Pathways

Another signaling pathway involved in immunosenescence is the mTOR pathway, which regulates the cell cycle and cell survival through the mTORC1 and mTORC2 complexes, both of which play vital roles in cellular metabolism and the immune response. However, decreased mTORC1 activity has been reported to disrupt autophagy and contribute to CD8^+^ T cell senescence in a manner dependent on p53 expression. Under normal physiological conditions, cytoplasmic p53 expression inhibits autophagy in an mTOR-dependent manner, whereas under stress conditions, nuclear p53 is upregulated through mTOR-independent mechanisms [121,122]. Furthermore, increased mTORC2 signaling has been observed in senescent CD4^+^ T cells with a deficient T-cell receptor (TCR) response, reducing their proliferative capacity and, consequently, their cell survival pathways [123].

Likewise, the JAK-STAT signaling pathway normally regulates responses to infection, immune tolerance, and tumor surveillance. However, with aging, it contributes to immunosenescence by promoting inflammaging through STAT3 hyperactivation, which triggers the SASP and increases IL-6 and IL-23 synthesis [124]. Similarly, repeated JAK-STAT activation alters hematopoietic stem cell (HSC) differentiation, favoring myeloid over lymphoid lineages, including neutrophils, macrophages, and senescent monocytes that increase proinflammatory cytokines such as TNF-α and IL-6, thereby promoting inflammaging [124,125,126,127,128].

Aging itself dysregulates neutrophil recruitment in response to aberrant chemokine signaling during a persistent inflammatory process that alters immune cell phenotype and function [125,126,127,128]. An increase in the β2-microglobulin component of the major histocompatibility complex (MHC-I) in plasma has been reported, leading to a pro-inflammatory monocyte phenotype linked to cognitive decline and impaired tissue regeneration in the adult brain [129,130].

Likewise, JAK1/2 hyperactivation promotes immunosenescence and reduced immune tolerance mediated by regulatory T cells (Tregs) through the SASP and through mutations in JAK3 and STAT5B, which generate immature T cells and natural killer (NK) cells that impair antitumor and anti-infectious responses [126,131]. Deficient IFN-I secretion has been reported. This molecule antagonizes cGAS-STING signaling primarily in senescent plasmacytoid dendritic cells (pDCs), which exhibit deficient production of interferon regulatory factor 7 (IRF7), resulting in reduced antigen presentation to T lymphocytes [132]. Another important proinflammatory factor is altered metabolism of saturated fatty acids and their increased plasma levels during aging, which promotes proinflammatory monocyte polarization and increases IL-10 and TGF-β1 production [133]. In addition, activation of the TLR2/6 pathway stimulates TNF-α and IL-6 synthesis and disrupts macrophage and monocyte polarization, reducing phagocytic capacity and allowing cellular waste to accumulate, thereby exacerbating inflammaging [133].

## 3. The Link Between Immunosenescence and Inflammaging

Immunosenescence is a complex process characterized by declining immune responsiveness, driven by factors including epigenetic remodeling, persistent antigenic stimulation, cellular senescence (the primary mediator of age-related cumulative cellular damage), thymic involution, and hematopoietic cell dysfunction that alters the B-to-T lymphocyte ratio. In the thymus, the precursor cell receptor complex (pre-TCR) reorganizes, and cellular metabolism shifts toward increased glycolysis [134,135,136,137,138], accompanied by mitochondrial dysfunction, decreased proteasome activity, and moderate levels of ROS that trigger lipid peroxidation chain reactions and induce apoptosis and autophagy in oxidatively damaged cells [139,140,141].

Immunosenescence is linked to inflammaging through a vicious cycle in which cGAS-STING-mediated damage to DNA and macromolecules reduces autophagy [36,142,143]. T-cell senescence increases aerobic glycolysis in response to increased expression of glucose transporters (GLUT) on the cell surface, driven by c-Myc-dependent upregulation of glycolysis-related genes [144]; this stress contributes to the inability to express key components of the electron transport chain (ETC) and to encode subunits of oxidative phosphorylation (OXPHOS), leading to increased ROS production and reduced proteasome activity, accompanied by decreased mitochondrial synthesis, which results in inefficient carbon and lipid metabolism [139,145].

Similarly, T-cell activity has been reported in the absence of acute infection, accompanied by the accumulation of senescent cells that express inhibitory receptors such as programmed cell death protein 1 (PD-1). When PD-1 binds its ligands (PD-L1 or PD-L2), it transmits inhibitory signals that suppress lymphocyte activation and proliferation. The KLRG1 receptor (killer cell lectin-related receptor, a marker of senescence) is a co-inhibitory molecule expressed primarily on natural killer (NK) cells and memory T cells [3,146]. Together, PD-1 and KLRG1 promote the secretion of proinflammatory mediators by increasing SASP activity [139,143,146,147,148].

Impaired neutrophil phagocytosis, marked by reduced antigen uptake and migration toward dendritic cells, hinders T-cell activation and reduces NK cell numbers and activity. Together, these factors weaken immune surveillance against pathogens, promote tumor formation, and disrupt the balance between pro-inflammatory and anti-inflammatory states [148,149,150,151,152,153]. Furthermore, late-differentiating T lymphocytes and myeloid cells derived from hematopoietic stem cells accumulate [9,143,148,149,150,151,152,153]. Consequently, immune dysfunction is not merely a passive consequence of aging but a key factor in the pathogenesis of diseases such as cancer, neurodegenerative disorders, cardiovascular diseases, and metabolic syndromes [4,24,25,26,27]. Age-associated immune remodeling alters leukocyte composition, impairs immune regulation, and sustains proinflammatory mediator production that contributes to inflammaging [2,28].

## 4. Alterations in Immunometabolism and Inflammation Resolution During Aging

During the activation of an immune response, immune cells transition from a quiescent metabolic state mediated by oxidative phosphorylation (OXPHOS), which generates 32 to 34 ATP molecules per glucose molecule and supports energy production, the synthesis of biomass precursors, and the synthesis of amino acids and lipids that meet the metabolic demands required for immune effector functions. Mitochondrial function is fundamental to the metabolic programming of T cells in type 2 immunity and the Th17-associated inflammatory response [2,28,154,155].

In this context, unlike in a young organism, chronic inflammation in aging also results from impaired mechanisms for resolving active inflammation. This includes the failure of specialized pro-resolving mediators (SPMs), lipid mediators that belong to a group of pro-resolving anti-inflammatory agents produced by innate immune cells in the inflammatory environment [156,157]. Two other families of bioactive lipids, lysoglycerophospholipids and sphingolipids, as well as endocannabinoids (eCBs), have also been described as initiators and resolvers of the inflammatory response. To this end, immune cells must constantly adapt their metabolic programming in response to changes in the microenvironment [158,159,160].

Disrupting the link between mitochondria-mediated cellular metabolism and immune functions through multiple pathways, including the tricarboxylic acid (TCA) cycle, oxidative phosphorylation (OXPHOS), and fatty acid and amino acid metabolism, is critical for maintaining the immunometabolic balance needed to resolve inflammation [156,157,158,159]. However, immunometabolic alterations, such as reduced fatty acid oxidation and mitochondrial dysfunction, can promote excessive inflammation. Similarly, ROS signaling can be detected as DAMPs, and mitochondrial ROS, together with the concomitant release of damage signals, are key execution nodes for mitochondria-targeted immune modulation. This modulation, through activation of SASP and the cGAS-STING, NF-κB, and NLRP3 pathways, exacerbates inflammaging [155,161].

Changes in energy metabolism shape the course of immune responses, from activation to long-term homeostatic adaptation. Metabolic programs adapt immune phenotypes to the microenvironment, thereby establishing thresholds for immune cell activation, effector response intensity, and differentiation pathways [162,163]. The metabolic-mitochondrial axis also influences immune function and inflammatory susceptibility through lipogenic pathways and the cholesterol/mevalonate axis, in which macrophage inflammatory polarization depends on mevalonate, the ionized form of mevalonic acid produced from HMG-CoA by HMG-CoA reductase [155,164].

Mevalonate mediates macrophage inflammatory polarization; persistent mevalonate activation increases acetyl-CoA and intermediate metabolites that promote histone acetylation and methylation, thereby altering Th17/Treg function and promoting M1 macrophage polarization (pro-inflammatory and release of lipid mediators (LM)). This process involves phospholipase A2 (PLA2), which cleaves phospholipids from cell membranes and releases arachidonic acid (AA). Upon oxidation, AA yields eicosanoids such as prostaglandins (PG), thromboxanes, and leukotrienes (LT) via the cyclooxygenase (COX) and 5-lipoxygenase (5-LOX) pathways, respectively [164,165,166]. However, immunosenescence in macrophages impairs their ability to adapt to the surrounding inflammatory microenvironment and reduces their pro-resolving (M2) phenotype, thereby maintaining a pro-inflammatory state with the release of cytokines, chemokines, and LM [46,167,168,169,170]. Under the “Phenotypic Continuum,” it is important to recognize the plastic capacity of macrophages to adopt intermediate phenotypes depending on their microenvironment, an aspect that remains poorly understood during the window of immunosenescence in aging [46,167,168,169,170].

Moss et al. [171] conducted an in vitro study using human monocyte-derived macrophages (MDMs) from young donors aged 18 to 30 and adults over 50. MDMs were stimulated for 24 h with LPS and IFN-γ. The study observed decreased phagocytosis, migration, and chemotaxis in macrophages from adults over 50 compared with those from young donors, accompanied by dysregulation of the target genes MYC and USF1 (upstream stimulatory factor 1), which encode transcription factors that regulate cell proliferation, differentiation, and apoptosis. Similarly, systemic accumulation of senescent macrophages can compromise BBB integrity, and within the brain parenchyma, senescent microglia maintain a pro-inflammatory profile that accelerates brain aging and enhances the progression of neurodegenerative disorders [172,173].

In addition, the AMPK (serine-threonine protein kinase) signaling pathway regulates energy metabolism and maintains cellular homeostasis by modulating and resolving oxidative stress. The AMPK pathway is critical for cell survival, growth, and autophagy by inhibiting mTOR, activating ULK1, and optimizing the NAD+/NADH balance through activation of the PGC-1α/SIRT1 signaling pathway, which maintains mitochondrial function and suppresses the inflammatory response by inhibiting the JAK-STAT, NF-κB, C/EBPβ, CHOP, and HIF-1α pathways [174,175,176]. Additionally, AMPKα1 supports immunological memory formation in CD8^+^ T lymphocytes by sensing glucose deprivation and suppressing mTORC1 activity [177].

Another regulatory mechanism of the inflammatory response is the melatonin signaling pathway, which, in addition to being a sleep inducer and a key molecule in the circadian cycle, modulates inflammation by inhibiting NF-κB and, consequently, the expression of the cytokines IL-1β, IL-6, TNF-α, and IFN-γ, while enhancing the anti-inflammatory cytokines IL-4 and IL-10 [178,179,180]. Furthermore, melatonin and its metabolites, such as N1-acetyl-N2-formyl-5-methoxykinuramine (AFMK) and N1-acetyl-5-methoxykinuramine (AMK), reduce ROS levels and increase the expression of antioxidant enzymes such as superoxide dismutase (SOD) and glutathione peroxidase (GPX), which protect mitochondrial DNA from oxidative damage and prevent the accumulation of SASP in immune cells, thereby positioning melatonin as a strategy against immunosenescence [178,179,180].

Melatonin affects the sirtuin (SIRT) signaling pathway, in which SIRT1 and SIRT6 have been shown to modulate the immune response through microRNA (miRNA)-dependent regulation of miR-146a, which acts on the Nrf2/NF-κB pathways [180,181,182]. Similarly, the melatonin signaling pathway has been shown to influence the response of CD4+/CD8+ T lymphocytes to a pathogen, NK cell cytotoxicity and the restoration of NK cell antitumor function, the regulation of autophagy, the modulatory activity of Treg/Th1/Th2 lymphocytes, and the polarization of macrophages toward an anti-inflammatory M2 phenotype [178,179,180]. In HSCs, SIRT3 plays a key role in regulating mitochondrial function, preserving genomic stability and delaying cellular senescence by increasing superoxide dismutase 2 (SOD2) activity [183,184,185]. Preserving these signaling pathways is essential to prevent immune decline and promote healthy aging.

## 5. Inflammaging and Blood–Brain Barrier Damage

Inflammation is an immune response to pathogen entry or tissue damage; depending on its severity or extent (local or systemic), it can induce metabolic and neuroendocrine changes that promote proinflammatory immune activity [186]. Inflammaging manifests systemically and is characterized by elevated serum levels of cytokines such as TNF-α, IL-6, IL-1β, and IL-8, as well as acute-phase proteins such as C-reactive protein (CRP) [2,3,62,187]. Chronic sterile inflammation has been described in the cardiovascular system (e.g., atherosclerosis) and the musculoskeletal system (e.g., osteoarthritis) [62]. In the brain parenchyma, this sterile or neurogenic inflammation is referred to as age-associated neuroinflammation or neuroinflammaging [62,143]; both processes are currently recognized as triggers of neurodegeneration in aging, a stage marked by morphofunctional alterations in the brain that manifest as a progressive decline in motor and cognitive functions, associated with decreased tissue plasticity and regeneration caused by immunosenescence [139].

In this context, systemic inflammation affecting the CNS has been described, often accompanying systemic infections of non-neurotropic origin, such as gastrointestinal, urinary tract, and respiratory infections [188]. Preclinical and clinical studies have shown that peripheral inflammation in response to viral [189] or bacterial infection, or associated with a preexisting comorbidity, is a common factor in the development and progression of CNS diseases, primarily Alzheimer’s disease (AD), Parkinson’s disease (PD), and epilepsy [190].

The BBB, a dynamic and complex interface between circulating blood and the CNS, comprises cerebral endothelial cells closely associated with pericytes and astrocyte foot processes, forming a neurovascular unit (NVU). The NVU is surrounded by the basement membrane and organized by extracellular matrix proteins crucial for its formation and integrity. Cerebral endothelial cells are connected by tight junctions (TJs). Occludin, claudins, and junctional adhesion molecules are the main TJ components that prevent proteins and small, lipid-insoluble molecules from entering the brain [190,191,192,193]. These TJs form a paracellular diffusion barrier; in turn, this compartmentalized structure comprising MBECs not only protects the brain parenchyma but also regulates the entry of immune cells from the circulation and controls their access to the CNS via the glia limitans [189,190,191,192,193]. Pericytes are contractile cells that respond to neuronal activity and regulate vasculogenesis, angiogenesis, neurovascular coupling, and neuroinflammation; they also regulate capillary blood flow, provide stability, and facilitate MBEC maturation, while handling phagocytosis of cellular debris and controlling BBB permeability. Additionally, the end-feet of perivascular astrocytes ensheath cerebral microcapillaries; they are responsible for regulating BBB permeability and the regeneration of TJs, recruiting new pericytes, and promoting water homeostasis via the aquaporin-4 (AQP4) protein, while also regulating processes such as angiogenesis, metabolic activity and inflammation in conjunction with perivascular macrophages and microglia [189,194,195,196].

During aging, structural changes linked to pericyte and MBEC senescence are marked by DNA damage, mitochondrial dysfunction, and increased expression of the tumor-suppressor genes p16Ink4a and p21Cip1/Waf1, which induce cellular senescence and SASP expression. This phenotype promotes proinflammatory and prothrombotic signaling cascades, including the release of IL-6, IL-8, TNF-α, intercellular adhesion molecule-1 (ICAM-1), vascular cell adhesion molecule-1 (VCAM-1), and MMP-2/9 (Figure 2) [197,198]. In turn, senescent MBECs exhibit reduced vasodilatory capacity due to decreased endothelial nitric oxide synthase (eNOS) activity and increased pro-oxidant molecules such as peroxynitrite and ROS, as well as leukocyte and platelet adhesion that damages the vascular endothelium; this impairs cerebral blood flow, leading to microvascular injury and thrombosis. Furthermore, low oxygen and nutrient availability in cerebral parenchymal cells, particularly neurons, triggers the formation of β-amyloid (Aβ) protein aggregates, an etiological factor that initiates the Aβ pathology associated with vascular senescence [199,200]. Additionally, senescence of pericytes, MBECs, and astrocytes contributes to neurovascular dysfunction, which directly affects BBB permeability and facilitates the entry of cells (such as T lymphocytes) and proinflammatory molecules into the cerebral parenchyma [201,202].

Under normal physiological conditions, immune surveillance requires circulating T lymphocytes to cross the BBB. They migrate through postcapillary venules under laminar flow and interact with adhesion molecules that are normally expressed at low levels on BMECs. During neuroinflammation, these molecules are upregulated (particularly ICAM-1 and VCAM-1 on the endothelial cells of postcapillary venules), recruiting additional T lymphocytes that can move against blood flow to reach tricellular junctions and undergo diapedesis across BMECs (Figure 2) [203,204].

Antigen-dependent signaling and adhesion pathways mediate T lymphocyte transit across BMECs, and mechanical damage or laminar shear stress can even activate these pathways. BMECs express only low levels of major histocompatibility complex class I (MHC-I), whereas MHC class II is expressed by specialized antigen-presenting cells (APCs) such as border-associated macrophages (BAMs), CNS-associated macrophages (CAMs), and dendritic cells (DCs) [204,205,206,207].

Adyn et al. [208] evaluated BMECs’ capacity to cross-present exogenous antigens on both the luminal and abluminal sides, in vitro and in vivo, using an ODC-OVA mouse model. They found that BMECs can take up, process, and present exogenous antigens to CD8+ T cells, independent of CD8+ T cell cytotoxic activity. Although ovalbumin (OVA) uptake by CNS macrophages in ODC-OVA mice limits antigen uptake and presentation at the BBB, this uptake is sufficient to reduce the migration speed of OVA-specific CD8+ T cells within cerebral microcapillaries. These findings indicate that neuroinflammation associated with MHC class I-restricted antigen presentation at the BBB can reduce CD8+ T cell entry into the brain parenchyma; however, this differs from severe pro-inflammatory cases, where high antigen accumulation can lead to BBB breakdown mediated by CD8+ T cells themselves.

Additionally, Chen et al. [209] reported, using single-cell RNA sequencing of hippocampal BMECs from aged mice, that arterial and venous BMECs undergo transcriptional changes during normal aging, specifically upregulation of signaling pathways related to innate immunity and oxidative stress responses, as well as an increase in MHC class I-associated transcripts in BMECs. This suggests that “inflammaging” may alter BMECs’ antigen-presentation capacity.

Inflammatory aging has been reported to compromise the integrity of TJs and gap junctions, allowing the entry of peripheral immune cells and proinflammatory molecules, including the cytokines IL-1β, IL-6, IL-9, IL-17, IFN-γ, TNF-α, and CCL2, which activate astrocytes and microglia [189,191,192]. BBB deterioration during aging is a major factor in the loss of structures such as the hippocampus, cerebral cortex, and corpus callosum [189,210]. A gradual decrease in the proteins that make up TJs has been observed [211], indicating that decreased claudin-5 expression marks BBB aging and that decreased claudin-1 is associated with vascular senescence [212,213,214].

Chronic systemic inflammation activates the MAPK signaling pathway and increases the expression of the adhesion molecules VCAM-1 and ICAM-1, leading to mitochondrial damage and ER stress in BMECs; it can also alter astrocyte function in BBB formation and maintenance by changing their processes [215]. Astrocyte activation by peripheral immune cells induces changes in morphology and VEGF-A secretion; this, in turn, activates the eNOS pathway in BMECs, reducing occludin and claudin-5 expression and thereby facilitating the entry of peripheral immune cells into the brain parenchyma [216,217].

In a transgenic mouse model expressing the amyloid precursor protein (APP), peripheral LPS injection increased BBB permeability, allowing infiltration of peripheral proinflammatory factors, such as IL-6 and TNF-α, into the brain parenchyma [216,217]. A damaged BBB contributes to Aβ plaque and neurofibrillary tangle formation in the brain, which are characteristic of AD [218]. Furthermore, systemic inflammation induced by intraperitoneal LPS in mice increased tau protein activity extending from the entorhinal cortex to the hippocampus via a microglia-independent pathway, suggesting that acute microglial activation in a mouse model of systemic inflammation and BBB damage may contribute to tau protein transmission [217,218]. This tau protein is not attenuated during microglial inhibition, suggesting a link to BBB damage induced by systemic inflammation [219,220].

During acute inflammation, TJs show reduced structural protein expression and altered conformation, increasing BBB permeability. This, in turn, increases expression of the adhesion molecules ICAM-1 and VCAM-1 on BMECs and pericytes, facilitating greater interaction with astrocytes and microglia [221]. Furthermore, pericytes express TNFα and IL-1β receptors, thereby increasing ICAM-1 expression and facilitating albumin-mediated immune cell migration. TNFα and IL-1β also activate hydrolases, such as GTPases, which reorganize the actin cytoskeleton and create large gaps between adjacent PC [222,223,224]. Furthermore, pericyte stimulation releases nitric oxide (NO), interleukins, and VEGF, which reduce claudin and occludin expression and increase BBB permeability [222,223,224]. A recent study in a mouse model of chronic social defeat stress (CSDS) developed a behavioral phenotype of social avoidance with increased anhedonia, correlating with increased inflammatory Ly6Chi monocytes and neutrophils and decreased blood B cells, similar to humans with major depressive disorder (MDD). BBB disruption and the influx of leukocytes, cytokines, and CCR2+ monocytes into the brain parenchyma lead to the production of factors such as MMP8, which alters synaptic plasticity in structures such as the nucleus accumbens and contributes to MDD and anxiety [225,226]. 

Peripheral leukocyte influx into the brain parenchyma lowers the threshold for neuronal excitability and triggers a neuroinflammatory response via microglia and the release of proinflammatory cytokines such as TNF-α, thereby increasing intracellular Ca^2+^ permeability and promoting neuronal hyperexcitability and epileptic seizures [225,226]. The accumulation of CCR2-expressing monocytes in the hippocampus increases IL-1β release, altering the seizure threshold and promoting BBB disruption [227]. Following *Status epilepticus* (SE), BBB damage plays a decisive role in increased monocyte recruitment to the meninges, promoting microglial activation and maintaining a persistent state of neuroinflammation [228]. Several conditions, such as acute myocardial infarction or hepatitis, that involve systemic inflammation also trigger CCL2-mediated recruitment of meningeal macrophages and neutrophils, promoting microglial activation and increasing the release of TNF-α in structures such as the thalamus and hippocampus [229,230,231], where these cells alter dendritic spine plasticity and increase the release of interferon-γ (IFN-γ) [232].

## 6. Neuroinflammaging and Neurodegenerative Proteinopathies

Neuroinflammation is a defense mechanism in response to an external pathogen, aimed at maintaining homeostasis within the CNS; this neuroinflammatory microenvironment can be mediated by endogenous factors that promote glial cell activation. However, when an inflammatory response is not resolved in timing and magnitude, chronic neuroinflammation can lead to neurodegeneration [233,234].

Neurons are postmitotic cells that do not enter the cell cycle; however, they can acquire senescence-associated phenotypes, including activation of cyclin-dependent kinase inhibitors (e.g., p16INK4a and p21CIP1) and morphological changes such as axonal degeneration and loss of dendritic spines [235]. This state is marked by SASP activation in response to recurrent cellular damage, intracellular stress, and impaired tissue repair. Astrocytes, microglia, and neurons promote an age-associated neuroinflammatory environment known as “neuroinflammaging” by releasing IL-6, IL-1β, TNF-α, chemokines such as CCL2 and CXCL8, TGF-β, VEGF, and MMP9. These mediators act both autocrinally and paracrinally, thereby sustaining cellular senescence that can spread to neighboring cells and contribute to a cycle of cellular dysfunction, oxidative stress, and mitochondrial damage. In turn, activation of signaling cascades such as NF-κB and cGAS-STING helps maintain a proinflammatory microenvironment that triggers proteinopathy development during pathological brain aging [234,235,236,237,238].

Additionally, immunosenescence of neural stem cells and NUV dysfunction, which together can activate the SASP in brain parenchyma cells [2,3,17,52], lead to increased ROS and decreased NADPH-dependent antioxidant activity. In these cells, glial cells such as astrocytes and microglia release TNFα, IL-6, and IL-1β, promoting a persistent inflammatory environment mediated by NF-κB signaling. This creates a cycle of mtDNA oxidative damage, reduced mitophagy, loss of homeostasis, and further cellular senescence that can affect both BMECs forming the BBB and brain parenchyma cells [187,236,237].

Neurons and microglia activated by SASP show overexpression of TLR2, TLR4, TLR5, and inflammasome-associated genes, including caspase-1, IL-18, and IL-1 [17,238,239,240,241]. Similarly, microglia and astrocytes acquire morphological features that compromise normal function and promote proinflammatory cytokine release, collectively contributing to the accumulation of misfolded proteins such as alpha-synuclein (α-SYN), characteristic of PD, and tau protein, characteristic of AD [13,25,242,243]. However, activation of TLR2 and TLR4 on the microglial surface increases proinflammatory activity and cytokine release but not phagocytic capacity (Figure 3) [244,245,246].

A key characteristic of senescent microglia is the loss of normal function. During aging, microglia shift from a homeostatic surveillance state to a dystrophic, chronically activated phenotype, marked by increased lysosomal load and reduced phagocytic capacity for both Aβ aggregates and cellular debris [246,247]. Furthermore, secretion of pro-inflammatory cytokines such as IL-1β, TNF-α, and IL-6, along with elevated ROS and nitric oxide (NO), sustains a persistent neurotoxic environment, a phenomenon primarily described in AD [248].

Furthermore, senescent astrocytes exhibit reduced expression of glutamate transporters, including excitatory amino acid transporters 1 and 2 (EAAT1/EAAT2), and of neurotrophic molecules such as brain-derived neurotrophic factor (BDNF) and glial cell line-derived neurotrophic factor (GDNF), thereby compromising neuronal support and exacerbating excitotoxic damage [249,250]. Recurrent SASP activity has been reported to alter synaptic signaling and plasticity by influencing glutamate uptake and calcium homeostasis [251]. IL-6 and TNFα, released via the SASP, suppress long-term potentiation and reduce dendritic spine density, leading to failed memory consolidation [251]. Additionally, senescent astrocytes maintain persistent SASP activation, driving increased expression of MMP-9/2, IL-8, and VEGF, which compromises the extracellular matrix (ECM) and disrupts the BBB, thereby sustaining a neuroinflammatory environment within the brain parenchyma [252,253]. Alongside maintaining SASP activity, increased pro-oxidant molecules disrupt remyelination by damaging oligodendrocyte precursor cells, causing white matter atrophy [254,255]. This results from prolonged SASP activity within the brain parenchyma, where ECM remodeling failure has been reported, altering the biomechanical properties of neural tissue and disrupting communication between neurons and glial cells [255,256].

Floden et al. [257] reported an in vitro model using microglia derived from C57BL/6 mice at 0 days postnatal, 2 to 4 months, 6 to 8 months, or 12 to 17 months. Reactive microglia from 6 months onward lose the ability to phagocytose Aβ fibrils but retain the ability to interact with them, maintaining a proinflammatory profile compared to microglia from early postnatal stages. This facilitates Aβ aggregation and sustains a proinflammatory profile. In addition, Aβ can act as DAMPs and activate the inflammasome via the TLR pathway, leading to sustained production of inflammatory cytokines such as IL-1β [258,259,260]. Immunosenescence and neuroinflammaging are also relevant in PD, where neuroinflammation has been linked to degeneration of dopaminergic neurons in the substantia nigra and is associated with increased expression of inflammatory mediators such as IL-1β and TNF-α [261,262].

Like microglia, astrocytes shift their metabolic profile during aging, keeping them chronically reactive. This shift involves a transition from aerobic glycolysis to oxidative phosphorylation, reducing lactate supply and causing an energy imbalance in neurons. This, in turn, reduces the Krebs cycle and alters the metabolic genes mdh1 and mdh2 [262,263]. Senescent astrocytes are characterized by mitochondrial fragmentation, reduced resistance to oxidative stress, and decreased levels of synaptogenic factors, which contribute to reduced dendritic spine density and, consequently, reduced neuroplasticity [263].

In a study using spatial transcriptomic analysis of brains from three groups of young, middle-aged, and elderly mice of both sexes, seven distinct transcriptomic regions were identified that showed upregulation of RNA or inflammatory molecules associated with microglial activation and astrogliosis, along with complement activation, myeloid cell infiltration, demyelination of tract fibers, and a reduction in white matter volume in the elderly group compared to the young and middle-aged [263]. White matter is considered an early site of inflammation due to its macromolecular, vascular, and cellular composition. Because white matter is lipid-rich, it forms lipid peroxides upon oxidation; it is also less vascularized, making it more susceptible to hypoxic damage. Oligodendrocytes or oligodendrocyte progenitor cells (OPCs) in fiber-tract regions have been reported to become senescent, release SASP, and attract microglia. This supports microstructural changes in the white matter associated with axonal degradation and demyelination and linked to neuroimmunosenescence [263,264,265,266].

Additionally, metabolic alterations and energy deficits in neurons disrupt ionic gradients and, consequently, synaptic transmission. This, in turn, makes neurons more susceptible to damage due to excessive release of proinflammatory cytokines such as IL-1β and TNF-α, ROS, and complement activation. These can be identified as DAMPs and locally activate the SASP, affecting structures such as the cerebral cortex and hippocampus [264,265,266]. Based on the above, inflammaging is suggested to be not only a consequence but also an accelerator of the aging process and the decline in brain function. However, how it initiates and progresses in a neurodegenerative disorder is not yet fully understood.

Öberg et al. [267] analyzed plasma and cerebrospinal fluid (CSF) samples from healthy young (25–30 years) and elderly (50–70 years) individuals, as well as from patients with PD, and observed an increase in IFN-I associated with aging rather than with NF-κB activity, which was a key characteristic distinguishing PD patients from healthy or young individuals. Additionally, a female PD patient was reported to have a gain-of-function mutation at G2019S, which confers gain-of-function kinase activity of LRRK2 (LRRK2GoF)3,6–8,25, associated with neuronal toxicity [267,268]. Similarly, IFNB1 transcripts increased, but TNF-α did not; after incubation with the inhibitor MLI-2, LRRK2 levels returned to those seen in healthy patients. These findings indicate that PD is characterized by an age-related, exaggerated IFN-I response [267,268].

Furthermore, in an in vivo study of young (3 months) and aged (12 months) Lrrk2GoF mice, we observed a markedly elevated, age-dependent IFN-I signature in plasma compared with wild-type (WT) mice. Lrrk2GoF mice also exhibited elevated levels of inflammatory monocytes (CD11b+Ly6C+) in the blood and increased expression of the leukocyte activation marker CD86. Similarly, inguinal white adipose tissue (iWAT) from Lrrk2GoF mice showed increased beta-galactosidase activity associated with senescence, suggesting that the key consequences in these mice are accelerated physiological aging and increased inflammaging [267]. In addition, using qPCR and bulk RNA-seq, we observed increased expression of IFN-I genes (Ifnβ1 and Ifnαs) and IFN-I response genes (ISGs) in neurons and microglia from aged Lrrk2GoF mice, which was associated with decreased age-dependent locomotor activity compared to WT mice. Similarly, intravenous injection of Evans blue into the tails of 3- and 12-month-old WT and Lrrk2 mice showed that Lrrk2GoF also compromises BBB integrity and reduces brain volume, suggesting that age-associated neuroinflammation contributes to this compromise [267,268].

Another important fact is that the IFN-I response occurs through TLRs on the cell membrane or through intracellular innate immune receptors, such as the cyclic GMP-AMP synthase (cGAS) enzyme [269,270,271,272,273]. These TLRs monitor the extracellular space for danger signals and transmit signals via TRIF/TICAM adaptors (adaptors containing a TIR domain that induce IFN-β production) [273]. As mentioned in previous sections, cGAS is an enzyme that, upon binding DNA within the cell, synthesizes cGAMP (cyclic GMP-AMP) from ATP and guanosine triphosphate (GTP). cGAMP signals through the STING adaptor to induce expression of immune genes, including those encoding IFN-I. Öberg et al. [267], by crossbreeding Lrrk2GoF mice with TRIF- or STING-deficient mice, reported that STING deletion completely reversed the elevated IFN-I response in Lrrk2GoF mice or cells, bringing it down to levels comparable to WT controls. This STING deficiency also reduced expression of cellular markers of inflammation in the microglia of aged mice, including CD86 and major histocompatibility complex class II (MHC-II), suggesting that Lrrk2GoF-induced inflammaging and the resulting neurodegeneration are primarily mediated by spontaneous activation of the STING-IFN-I axis.

Furthermore, in humans, aging was associated with increased accumulation of extracellular vesicles (EVs) containing DNA in the blood and CSF, a phenomenon more pronounced in patients with PD. When incubated with bone marrow-derived macrophages (BMDMs), EVs isolated from PD patients induced a STING-dependent IFN-I response. These findings indicate that aging and the LRRK2GoF variant converge to impair endolysosomal clearance, promoting the systemic accumulation of DNA-laden EVs. Additionally, intraperitoneal administration of the STING agonist DMXAA in mice confirmed that peripheral STING activation can alter the BBB and cause neurological deficits [267]. These findings broaden the perspective on the origin of PD, shifting it from an inflammatory disorder confined to the brain to one with peripheral origins.

Another important factor is the gender-specific divergence in neuroimmunosenescence-related changes, mediated by hormonal regulation and epigenetic factors throughout life, that contribute to the development of neurodegenerative disorders such as AD. AD shows a higher prevalence of cognitive decline in women compared to PD, which is more common in men [274,275]. This has been attributed to the modulatory and neuroprotective activity of steroid hormones on glial cells that make up the BBB, including microglia and astrocytes, as well as on neurons during early and young adult stages [276,277,278].

However, the neuroimmune dysregulation observed in aging occurs alongside changes driven by immunosenescence, characterized by declines in the steroid hormones estrogen, progesterone, and testosterone, and associated with chronic inflammatory phenotypes, neurovascular dysfunction, and metabolic abnormalities [279,280]. Sex-specific differences in responses to aging have been described, primarily regarding the female immune system, where higher levels of CD4+ T lymphocytes and a higher CD4/CD8 ratio have been identified; greater SASP activation in monocytes and increased interferon production by pDCs have been observed, in contrast to what is observed in men, who exhibit a greater accumulation of senescent CD8+ T lymphocytes [281,282,283,284].

Furthermore, in women, decreased estrogen promotes Th1 pro-inflammatory responses, whereas higher levels promote Th2 humoral immunity and antioxidant activity by inhibiting ROS and upregulating antioxidant systems such as SOD and GPX [285,286]. In men, immunosenescence progresses more slowly, with lower levels of inflammaging but higher cardiovascular and metabolic risk than in women [287,288,289]. However, women show more intense microglial reactivity, with greater phagocytic activity and a more pro-inflammatory profile, primarily in the hippocampus [289,290,291].

## 7. Molecular and Structural Biomarkers of Neurodegenerative Proteinopathies

Another hallmark of brain aging and neurodegeneration is the decline in the ability to maintain proteostasis, which is reflected in protein aggregation in neurodegenerative diseases; however, this decline is not yet fully understood. The presence of specific protein aggregates, such as Aβ and phosphorylated tau in AD, α-SYN in neurons for PD, α-SYN in oligodendrocytes for multiple system atrophy (MSA), and TAR DNA-binding protein 43 (TDP-43) for amyotrophic lateral sclerosis (ALS) and a type of frontotemporal dementia (FTD), is a hallmark of neurodegenerative diseases and an indicator of neurodegenerative proteinopathies [3,28,242,292]. 

In neurons, the soma, where the Golgi apparatus resides, is the primary site of transcription for transcripts encoding many components of the degradation machinery; this may limit the extent to which distal compartments maintain and adapt synaptic protein proteostasis [293,294]. Synaptic proteins require a degradation apparatus that locally regulates their composition via the ubiquitin-proteasome system (UPS), which degrades ubiquitin-tagged proteins [295]. Another degradation pathway transports cargo to catalytically active lysosomes, the final destination for endosomes, autophagosomes, and phagosomes; as a result, lysosomes maintain an acidic pH that optimizes soluble hydrolase activity [296].

Distal autophagosomes, responsible for somatic macroautophagy, must travel from the synapse to the soma, where autophagosome biogenesis occurs, and then fuse with lysosomes to degrade protein aggregates in various neurodegenerative diseases [297,298]. This local degradation mechanism is fundamental in dendrites and synapses, where elevated levels of the 20S proteasome alpha-1 subunit (PSMA1), which degrades damaged, misfolded, or unnecessary intracellular proteins, negatively correlate with its mRNA, particularly in distal dendrites [299,300]. Therefore, because the neuronal soma provides the structural and degradative capacity of the proteasome, it may also limit the adaptation of distal structures, such as dendrites, to proteostasis during proteotoxic stress, making neurons vulnerable to oxidative stress during aging, a primary risk factor for more than 50 diseases involving abnormal protein deposition (proteinopathies) [300,301,302].

Furthermore, epigenetic changes in glial cells have been described as a key link between neuroinflammation and neurodegeneration, with common epigenetic hallmarks of neurodegeneration, including DNA methylation, histone modifications, and microRNAs, that predispose individuals to AD [303,304]. Similarly, methylation of the α-SYN and tau genes, which are associated with microtubules, may contribute to PD development [305]. In this regard, histone H3 phosphorylation (Ser10) and histone H3 acetylation (Lys14) in microglia have been observed more frequently in patients with AD. These modifications are associated with increased production of IL-1β, IL-6, and TNF-α, as well as increased NF-κB activity, reinforcing the vicious cycle of inflammaging and immunosenescence that promotes neuroinflammation, mitochondrial dysfunction, neuronal damage, and cell death [306,307,308,309,310,311,312,313].

Although brain aging is not uniform, Chaudhari et al. [314] used magnetic resonance imaging (MRI) to map the brains of 14,748 cognitively normal (CN) subjects from multiple centers; local brain age (LBA) was estimated at the voxel level (the three-dimensional volume equivalent of a pixel in 2D images), revealing spatial patterns of aging in the frontal and temporal cortex that were similar in subjects with atypical aging and those with AD and that correlated with cognitive impairments associated with the progression of neurodegeneration. This finding identifies inflamaging as a key factor in immune, glial, neuronal, and vascular dysfunction [315]. Among these actors, IL-6 signaling has been linked to amyloid precursor protein (APP), tau protein, and vascular dysfunction; Aβ can stimulate IL-6 production in glial cells, creating positive feedback loops of inflammation [316,317,318,319]. Furthermore, phosphorylated tau protein self-assembles into amorphous aggregates that activate the TLR4 pathway in macrophages, thereby elevating IL-6 levels. This demonstrates that tau protein can act before IL-6 induction in microglia, contributing to AD progression [320].

Similarly, in PD, elevated IL-6 levels in the CSF and peripheral blood have been correlated with disease severity [321,322]. These findings establish that misfolded α-SYN can activate microglia to release IL-6 and that IL-6 trans-signaling promotes iron sequestration (increased uptake and reduced export), leading to intracellular iron accumulation and oxidative stress that damage dopaminergic neurons in the substantia nigra [323]. Similarly, in Huntington’s disease (HD), the striatum shows a marked increase in IL-6, accompanied by overactivation of monocytes, macrophages, and microglia in both animal models and patients [324,325].

IL-6 overexpression is linked to astrocyte dysfunction via the JAK/STAT3 pathway [326] and to increased glutamate-mediated excitotoxicity [327,328]. Another key role of IL-6 in neurodegeneration has been described in multiple sclerosis (MS), where elevated levels of IL-6 and s1L-6R have been reported in the serum and CSF of MS patients [329,330], correlating with the severity of inflammatory lesions and reduced synaptic plasticity [331]. Notably, IL-6 activity has been implicated in differentiating pathogenic CD4+ T lymphocytes toward Th17/Th1-associated mechanisms, which may limit Treg induction and promote autoimmune activity [332,333,334]. Similarly, dysregulated IL-6 signaling alters microglial phagocytic activity, thereby facilitating the progression of proteopathies and the recruitment of peripheral immune cells [335,336,337]. 

A major challenge is identifying a specific, universal marker of senescence in the CNS. Because the senescent phenotype is inherently complex and spans many cell types, the currently accepted strategy is a multi-marker approach. This approach has led to a wide variety of phenotypes being classified as senescence markers in neurons, some of which are observed in states other than senescence; however, because aging is heterogeneous, these markers have been characterized across multiple mechanisms driving cellular arrest, particularly in pathologies such as Alzheimer’s disease (AD). One of the most common markers of neuronal senescence is the expression of cyclin-dependent kinase inhibitors, including p21, p16, and p19. p21 is expressed in senescent neurons derived in vitro from iPS cells from individuals with Parkinson’s disease, spinal cord injury, and hyperinsulinemia. Markers of DNA damage are also prominent in neuronal senescence, as shown by the DSB marker phosphorylated histone H2AX (γ-H2AX), changes in nuclear size or structure, epigenetic remodeling, and the formation of senescence-associated heterochromatin foci [338].

Similarly, transcriptomic and proteomic analyses have identified SASP activity in specific states of cellular arrest, primarily in senescent astrocytes, which downregulate genes encoding glial fibrillary acidic protein (GFAP) and S100β proteins while increasing IL-6, IL-8, and MMP-3. In contrast, senescent microglia show increased CCL2, CXCL10, and complement protein expression [339,340]. Senescent microglia are characterized by dysregulated pro-inflammatory activity and reduced phagocytic activity, which facilitate Aβ accumulation and dystrophic neurite formation. Persistent SASP secretion promotes neuroinflammation, induces senescence in astrocytes and surrounding neurons, and facilitates the recruitment of peripheral immune cells that penetrate the brain parenchyma through the damaged BBB [341].

Researchers have also identified several molecules proposed as senescence markers in astrocytes, microglia, oligodendrocytes, and neurons. Expression of p16 and p21 has been linked to senescence in astrocytes, microglia, endothelial cells (ECs), and oligodendrocyte precursor cells (OPCs) [342,343,344]. Senescence-associated β-galactosidase (SA-β-gal), a marker of senescence-linked lysosomal expansion, has also been reported in astrocytes and microglia in murine models of AD; however, SA-β-gal expression is not exclusive to cellular senescence, as it can also be induced by cellular stress [345,346,347,348]. Another widely studied marker is the protein p16INK4a (CDKN2A), which induces cell cycle arrest in senescent cells by inhibiting CDK4/6 and subsequent retinoblastoma protein (pRB) phosphorylation [2,3,349,350].

Similarly, senescence-associated heterochromatin foci (SAHF) have been identified using DAPI staining, which labels them as dense, punctate nuclear structures associated with chromatin remodeling during proliferation arrest [351,352]. Another characteristic of senescent glial cells and endothelial cells (ECs) is the loss of laminin B1, a nuclear envelope protein, as well as the presence of classic lipofuscin aggregates, which bind oxidized proteins and lipids, primarily in neurons and glial cells [353,354,355].

Likewise, senescent microglia are characterized by increased p16 expression and dystrophic morphology with fragmented processes [356]. Additionally, microglia in the white matter are reported to be the primary cell type undergoing cellular senescence in the CNS during aging, and microglia/macrophage-specific elimination of p16INK4a attenuates neuroinflammatory phenotypes in an experimental autoimmune encephalomyelitis (EAE) model in mice [355]. Although these markers can identify morphological changes in glial cells and neurons, their activation is not typically specific to cellular senescence, as stressors can induce their expression, making them non-specific [353,354,355].

Recent studies have identified blood biomarkers with predictive potential, particularly for AD, with the aim of distinguishing it in a timely manner from conditions such as FTD and other primary psychiatric disorders. These studies measure plasma levels of tau protein phosphorylated at position 217 (p-tau217) and the neurofilament light chain (NfL) and correlate them with imaging studies as specific markers for detecting AD [356,357,358]. NfL has been described as a marker of acute neuronal damage and axonal degeneration that allows differentiation between a neurodegenerative disorder and a psychiatric disorder [357,358]. Another plasma marker is GFAP, which indicates astrocyte reactivity and neuroinflammatory activity in various neurodegenerative diseases. However, its expression remains controversial because its specificity is unclear, making it difficult to correlate its expression with clinical manifestations across AD, other neurodegenerative disorders, and psychiatric disorders compared with the expression of the p-tau217 and NfL proteins [358,359,360].

In ALS, FTD, and age-related TDP-43 encephalopathy with limbic predominance (LATE), characterized by the abnormal cytoplasmic localization and aggregation of RNA-binding proteins such as TAR-43 (TDP-43), which is found in 97% of patients with ALS, 50% of patients with FTD, and 100% of LATE cases, has also been identified in some cases of AD, PD, and HD, contributing to the heterogeneity among these proteinopathies, the cause of which is not yet fully understood [361,362,363,364,365].

TDP-43 performs important functions in gene expression and RNA metabolism, including transcription, translation, splicing, and mRNA stability; loss of TDP-43 causes abnormal splicing, which leads to neurodegeneration [366]. Abnormal TDP-43 localization has been reported to induce the inclusion of cryptic exons in genes that regulate neuronal function, such as UNC13A and STMN2, leading to decreased expression of the proteins encoded by these genes [367]. Furthermore, TDP-43 plays a key role in cellular stress responses by regulating mRNA levels, apoptosis, axonal transport, and ribonucleoprotein transport [368,369]. It also plays a crucial role in repairing double-strand breaks (DSBs) in DNA via non-homologous end joining and in resolving R-loops, both processes implicated in the pathology of ALS and FTD [370,371]. The loss of nuclear TDP-43 protein indicates a loss-of-function (LOF) phenotype associated with its accumulation in the cytosol, which leads to neuronal toxicity, the accumulation of DNA breaks, exacerbated neuroinflammatory responses, senescence, and neuronal dysfunction in the CNS, with pathophysiological characteristics similar to those observed in ALS [371,372].

Additionally, loss of RNA splicing has been correlated with the senescence marker p16, which is associated with TDP-43 dysfunction. Furthermore, loss of RNA splicing has been correlated with the senescence marker p16, which is associated with TDP-43 dysfunction. Likewise, alterations in the TDP-43 protein have been described in both normal aging and age-related neurodegenerative disorders [373,374]. This demonstrates that SASP activity—which converges on neuroinflammation—is not only associated with aging but is also expressed in cells and tissues affected by various diseases linked to the aging process [267,373,374].

The PD-1/PD-L1 pathway has been described in the context of immune control and tolerance, primarily in cancer studies, where PD-1 (CD279) and its ligands, PD-L1 and PD-L2, can regulate T-cell activation and proliferation. Structural analysis has revealed that PD-1 shares 15% sequence similarity with CD28, 20% with CTLA-4, and 13% with the inducible T-cell co-stimulatory factor [375]. This allows PD-1 to inhibit innate and adaptive immune responses. It is expressed on T lymphocytes and can be activated by transcription factors such as nuclear factor of activated T cells (NFAT), NOTCH, forehead box O1 (FOXO1), and interferon regulatory factor 9 (IRF9), which initiate its transcription [376,377].

However, every modulatory molecule has two sides, and PD-1 activity can be both beneficial and harmful. It has been reported that PD-1 can also promote the replication of malignant cells, and its expression, along with that of PD-1/PD-L1, may play a role in the aging process. Increased PD-1 levels have been reported in tissues such as the bone marrow, heart, kidney, liver, lung, cerebellum, and spleen [378], alongside increased expression of p16 and p21, markers of cellular senescence [379]. Senescent cells can secrete exosomes with immunosuppressive properties that carry proteins such as PD-L1 [380].

Similarly, PD-L1 activity is mediated by inflammatory factors secreted by these senescent cells, including the JAK/STAT3 pathway; therefore, increased PD-L1 expression correlates with higher levels of proinflammatory factors [381]. Wang et al. [381] reported that treating aged mice with an anti-PD-1 antibody reduced the number of senescent cells and PD-L1-positive cells in the liver, indicating that senescent cells could not evade immune elimination following immunotherapy. It has been reported that PD-1 expression increases in human senescent T cells and leads to a decrease in apoptosis induced by PD-L1-positive stromal cells [382,383]; these stromal cells, which appear with aging or in age-related diseases, possess a dual defense mechanism against immune elimination: on the one hand, increased PD-L1 expression strengthens their immune defense against cytotoxic immune cells; on the other hand, senescence or T-cell exhaustion associated with aging leads to increased expression of PD-1, which impairs immune cell function, particularly that of CD8+ cytotoxic T lymphocytes and NK cells. This shows that senescent cells can protect themselves from immune surveillance and subsequent elimination, leading to their accumulation in tissues with aging [384].

During healthy aging, changes occur in the BBB, and these changes can be further exacerbated in neurodegenerative disorders such as AD, PD, diabetic retinopathy, epilepsy, ALS, MS, and HD. Therefore, strategies to prevent these conditions have focused on maintaining the BBB through therapeutic interventions, based on the premise that restoring the BBB, at least to some extent, could delay disease pathogenesis. Strengthening the barrier could reduce the negative effects of inflammation; alternatively, in neurodegenerative disease, maintaining BBB integrity could delay disease progression and neurodegeneration [385]. Treatments have focused on two groups: pharmacological treatments and, more recently, gut-derived microbial metabolites, given their potential to modulate the BBB. Interestingly, changes in the gut microbiota have been associated with changes in the brain and with conditions such as neurodegenerative disorders. This connection between the gut microbiota and the brain is known as the gut–brain axis, a field that has attracted growing interest over the past two decades. Many categories of microbial metabolites, including short-chain fatty acids (SCFAs), bile acids, neurotransmitters, and other bioactive molecules produced in the gut, influence brain signaling. Few studies have examined their direct interactions with the BBB, leaving many opportunities to explore the physiological effects of these metabolites [386].

Therefore, although aging is a universal, progressive, and heterogeneous deterioration, with each organism aging differently, evidence suggests a delay in age-related decline in brain function compared with that of peripheral organs [267]. Although physiological and structural deterioration may unfold differently, these processes converge to generate immunosenescence and inflammaging, which are not only consequences of aging per se but also factors that accelerate these mechanisms and correlate with the rate of progression and severity of brain deterioration. Although new preclinical and clinical findings continue to emerge on the factors that trigger the onset of immunosenescence and inflammaging and their culmination in neurodegeneration, the molecular mechanisms involved are not yet fully understood [267,268].

## 8. Conclusions

The blood–brain barrier (BBB) is essential for protecting the brain, and its disruption has been linked to neurodegenerative diseases, including multiple sclerosis, ALS, Alzheimer’s disease, diabetic retinopathy, epilepsy, and stroke. Therefore, approaches to preventing these conditions may focus on maintaining the BBB through therapeutic strategies that either open it to deliver drugs to the CNS or reinforce it to slow the progression of neurodegenerative diseases.

Inflammaging and neuroinflammaging, two forms of persistent chronic inflammation, one systemic and the other within the brain parenchyma, play a central role in the onset and progression of neurodegenerative disorders. Aging is heterogeneous and marked by epigenetic alterations that promote cellular senescence, activate SASP, and trigger signaling cascades involving cGAS-STING, C/EBPβ, NF-κB, and mTOR, which converge into a vicious cycle of immunosenescence and inflammaging. This cycle is further fueled by alterations in innate and adaptive immunity, failure of inflammatory resolution mechanisms, and BBB damage.

The blood–brain barrier acts as a communication interface between the immune system and the brain parenchyma. Glial cells and MBECs can produce pro-inflammatory mediators that alter this neuroimmune communication, maintaining a neuroinflammatory environment and persistent microglial activation that drives progression of proteinopathies. Consequently, preserving the blood–brain barrier (BBB) constitutes a key protective strategy against the recruitment and continuous entry of peripheral immune cells into the brain parenchyma, as well as a means of modulating the proinflammatory reactivity of glial cells. However, despite recent studies describing the direct involvement of mitochondrial damage in SASP activation and related signaling pathways, the relationship between immunosenescence mechanisms and BBB damage remains poorly understood. These mechanisms, in turn, offer opportunities to develop preventive and therapeutic strategies that modulate immunosenescence and to identify peripheral biomarkers that reflect the state of the neuronal microenvironment and its selective vulnerability in specific neuronal subtypes during the development and progression of neurodegenerative diseases. Therefore, protecting the BBB during aging could serve as a preventive strategy against neurodegeneration and as a modulator of neuroinflammation.

## Figures and Tables

**Figure 1:**
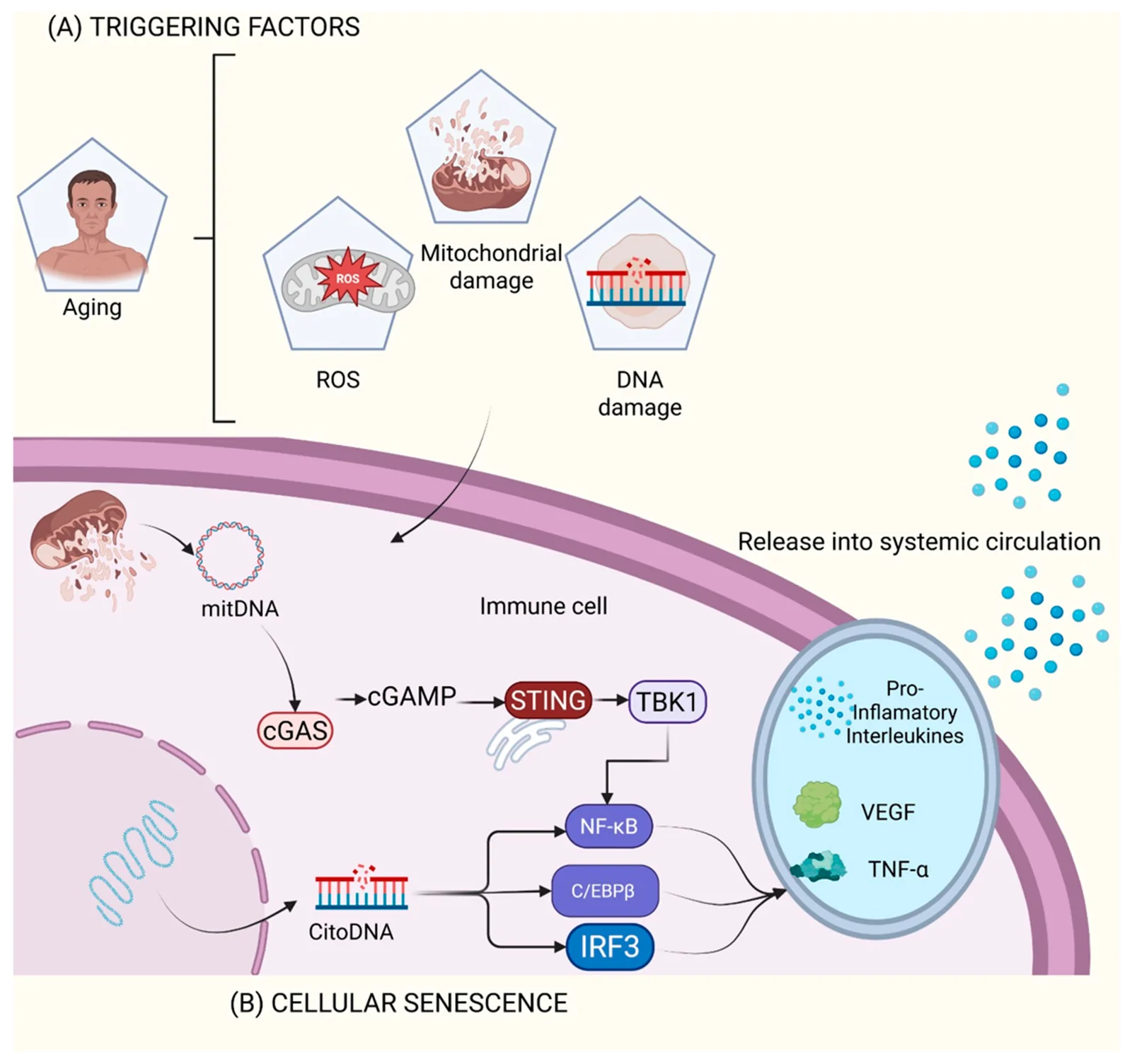
Cellular senescence and inflammaging. (**A**) Factors associated with aging, including DNA damage, oxidative stress, reactive oxygen species (ROS), mitochondrial dysfunction, and impaired proteostasis, promote cellular senescence. (**B**) Cellular damage stimulates the release of mitochondrial DNA (mtDNA) and cytoplasmic DNA (cytoDNA), which act as DAMPs and activate the cGAS–cGAMP–STING–TBK1 pathway, leading to activation of NF-κB, C/EBPβ, and IRF3 and to expression of proinflammatory mediators such as IL-1β, IL-6, TNF-α, and VEGF.

**Figure 2:**
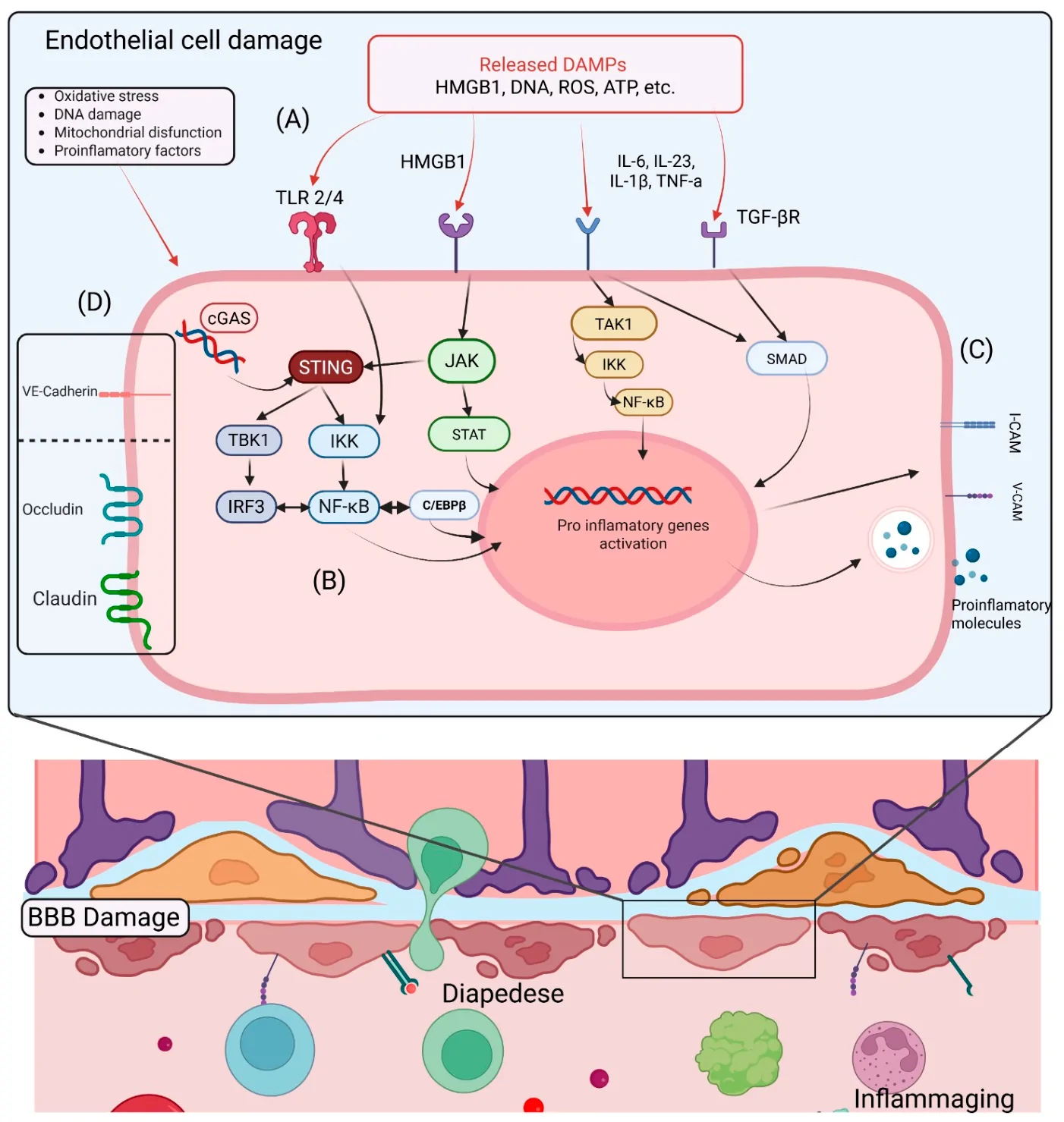
Endothelial damage activated by inflammaging. Endothelial damage activated by persistent chronic inflammation. (**A**) DAMPs (HMGB1, DNA, ROS, and ATP) and proinflammatory cytokines (IL-6, IL-23, IL-1β, and TNF-α) activate receptors such as TLR2/4, JAK, and TGF-βR. (**B**) These signals activate the cGAS–STING–TBK1–IRF3, IKK–NF-κB, JAK–STAT, and TGF-β–SMAD pathways, whose transcription factors (IRF3, NF-κB, STAT, and SMAD) induce proinflammatory genes. (**C**) The response increases the release of inflammatory mediators and the expression of adhesion molecules ICAM-1 and VCAM-1, promoting leukocyte adhesion and diapedesis. (**D**) Oxidative stress, DNA damage, and mitochondrial dysfunction alter endothelial junction proteins, including occludin and claudins, and the adherents junction protein VE-cadherin, compromising BBB integrity and promoting dysfunction.

**Figure 3:**
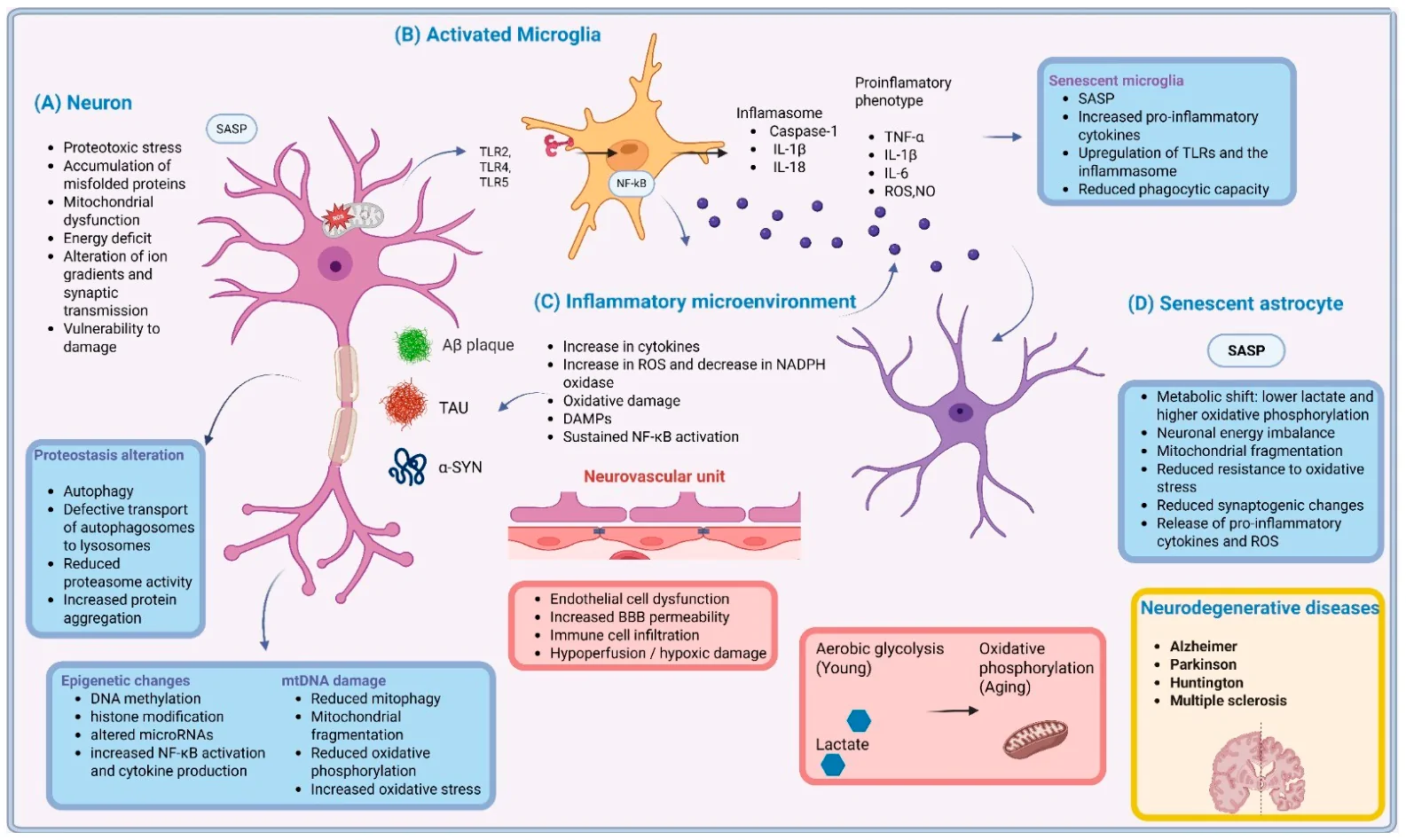
Neuroinflammaging and neurodegeneration. Cellular and molecular mechanisms of neuroinflammaging and immunosenescence associated with neurodegeneration. (**A**) Neurons: Aging promotes oxidative stress, mitochondrial dysfunction, impaired proteostasis, and the accumulation of misfolded proteins, including Aβ, tau and α-SYN. (**B**) Activated microglia: Activation of TLR2, TLR4, and TLR5, along with the inflammasome and NF-κB, promotes the release of proinflammatory cytokines, including TNF-α, IL-1β, and IL-6, and reduces phagocytic capacity. (**C**) Inflammatory microenvironment: Increased levels of cytokines, ROS, and DAMPs promote oxidative damage, mitochondrial dysfunction, and sustained inflammation. (**D**) Reactive/senescent astrocytes: These cells exhibit metabolic alterations, mitochondrial fragmentation, reduced resistance to oxidative stress, and the release of SASP, all of which contribute to the loss of neuronal plasticity and homeostasis. Taken together, these processes, along with neurovascular dysfunction, oligodendrocyte senescence, and demyelination, establish a vicious cycle of immunosenescence–inflammaging–neurodegeneration, associated with diseases such as Alzheimer’s, Parkinson’s, Huntington’s, and multiple sclerosis.

## Data Availability

No new data were created or analyzed in this study. Data sharing is not applicable to this article.

## Abbreviations

AD	Alzheimer’s disease
AKT	protein kinase B
AMPK	Serine-threonine protein kinase
APC	Antigen-presenting cells
DNA	deoxyribonucleic acid
AP-1	activator Protein-1
α-SYN	alpha-synuclein
ATM	ataxia telangiectasia mutated
BBB	blood–brain barrier
BL	B lymphocytes
BRD4	bromodomain-containing protein 4
C/EBPβ	CCAAT/enhancer-binding protein beta
CCF	cytoplasmic chromatin fragments
cGAMP	cyclic guanosine monophosphate-adenosine monophosphate
cGAS	cyclic GMP-AMP synthase
cIAP1	cellular inhibitor of apoptosis 1
cytoplasmic DNA	cytoplasmic deoxyribonucleic acid
CLR	C-type lectin receptors
CMN	neural stem cells
CNS	central nervous system
COX-2	cyclooxygenase-2
DAMPs	damage-associated molecular patterns
DNases	deoxyribonucleases
dsDNA	double-stranded DNA
E26	erythroblast-transforming factor
EC	endothelial cells
BMECs	Brain microcapillary endothelial cells
EGF	epidermal growth factor
NAFLD	non-alcoholic fatty liver disease
ELKS	Structural and regulatory protein
ER	endoplasmic reticulum
ERK	MEK phosphorylates and activates ERK
ETS family	the largest group of transcription factors
FOXO	Forkhead O box
G9A (EHMT2)	an enzyme responsible for the mono- and dimethylation of lysine 9 of histone H3 (H3K9)
G-CSF	granulocyte colony-stimulating factor
GH	growth hormone
GM-CSF	granulocyte-macrophage colony-stimulating factor
GR	glucocorticoid receptor
GTPases	hydrolase enzymes
HIF-1α	alpha subunit of hypoxia-inducible factor 1
HMGB1	high-mobility group box protein 1
HSP90	90 kDa heat shock protein
IAI	apoptotic innate immunity
ICAM-1	intercellular adhesion molecule 1
IFN-γ	interferon gamma
IGF	insulin-like growth factors
IGFBP-1	insulin-like growth factor-binding protein 1
IGFBP-2	insulin-like growth factor-binding protein 2
IL	interleukin
ILs	interleukins
IRF3	interferon regulatory factor 3
ISG	interferon-stimulated genes
JAK	Janus kinase
JNK	c-Jun N-terminal kinase
KLRG1	killer cell lectin-type receptor G1
LPS	lipopolysaccharide
Lys63	the amino acid lysine at position 63
MAPK	mitogen-activated protein kinases
MCP	monocyte chemotactic protein
MEK	RAF phosphorylates and activates MEK
MIP-1α	macrophage inflammatory protein-1
mtDNA	mitochondrial deoxyribonucleic acid
MKK3	mitogen-activated protein kinase 3
MKK6	mitogen-activated protein kinase 6
MLL1	lysine methyltransferase, also known as KMT2A
MMP1	matrix metalloproteinase 1
MMP12	matrix metalloproteinase 12
MMP3	matrix metalloproteinase 3
MMPs	matrix metalloproteinases
MTOR	Mechanistic target of rapamycin
mRNA	messenger ribonucleic acid
Mφ	macrophages
NEMO	NF-κB-modulating ubiquitin-binding protein
NF-κB	nuclear factor kappa B
NK	natural killer cells
NOD (NLR) type receptors	intracellular pattern recognition proteins that detect pathogens and stress signals within the cell
NOS	nitric oxide synthase
NOS2	nitric oxide synthase 2
NO	nitric oxide
OXPHOS	Oxidative phosphorylation
p38 MAPK	p38 mitogen-activated protein kinase
PAMPs	pathogen-associated molecular patterns
PC	pericytes
PD	Parkinson’s disease
PI3K	phosphoinositide 3-kinase
pre-TCR	precursor T-cell receptor
PRR	pathogen recognition receptors
PSMA1	20S proteasome subunit alpha 1
RAF	RAS activates the kinase RAF
RAGE	receptor for advanced glycation end products
RAS	It is a small protein in the cell membrane that receives signals from growth factors
RDA or DDR	DNA damage response
RIG-I-type receptors	cytosolic sensors of the innate immune system that detect viral RNA inside the cell
RIP1	receptor-interacting serine/threonine kinase 1
RIP1	receptor-interacting serine/threonine kinase.
ROS	reactive oxygen species
RTK	tyrosine kinase receptors
SASP	senescence-associated secretory phenotype
SMAD	intracellular protein chains that transmit chemical signals from the cell surface to the nucleus
STAT3	Signal Transducer and Activator of Transcription 3
STING	stimulator of interferon genes
TAB1	constitutively binds to the N-terminal end of TAK1 and is essential for its activation via autophosphorylation
TAB2 and TAB3	homologs that recognize polyubiquitin chains linked at Lys63
TAB4	additional adaptor that stimulates the TAK1-
TCA	Tricarboxylic acid
TAK1	adaptor proteins known as TAB
TAK1	kinase 1 aa
TBK1	TANK-binding kinase 1
TGF-β	transforming growth factor beta
Th	helper T cells
TIMP-1	tissue inhibitor of metalloproteinases
TJs	tight junctions
TL	T lymphocytes
TLRs	Toll-like receptors
TMEM9B	TMEM9 Domain Family Member B
TNF-α	tumor necrosis factor alpha
TRAF	tumor necrosis factor receptor-associated factors
TRAF6	tumor necrosis factor receptor-associated factor 6
Treg	regulatory T cells
VCAM-1	vascular cell adhesion molecule 1
VEGF	vascular endothelial growth factor
VEGF-A	vascular endothelial growth factor A
ZO1	zonula occludens-1
